# Pain-free hospital implementation: a multidimensional intervention to improve pain management at Wallaga University Referral Hospital, Nekemte, Ethiopia

**DOI:** 10.1186/s13104-024-06685-z

**Published:** 2024-01-18

**Authors:** Gedefa Bayisa, Kebena Limenu, Nemomsa Dugasa, Bikila Regassa, Muhamedamin Tafese, Mulugeta Abebe, Indalkachew Shifera, Diriba Fayisa, Habtamu Deressa, Asefa Negari, Amsalu Takele, Temesgen Tilahun

**Affiliations:** 1 Quality Unit, Wallaga University Referral Hospital, Nekemte, Ethiopia; 2 Oncology Unit, Wallaga University Referral Hospital, Nekemte, Ethiopia; 3 Department of Anesthesia, School of medicine, Wallaga University, Nekemte, Ethiopia; 4 Nursing Unit, Wallaga University Referral Hospital, Nekemte, Ethiopia; 5 Department of Emergency and Critical Care, School of medicine, Wallaga University, Nekemte, Ethiopia; 6 Department of Surgery, School of medicine, Wallaga University, Nekemte, Ethiopia; 7 Department of Obstetrics & Gynecology, School of medicine, Wallaga University, Nekemte, Ethiopia

**Keywords:** Pain-free hospital, PDSA cycle, Wallaga, Ethiopia

## Abstract

**Objective:**

This quality improvement project is aimed to increase pain free hospital implementation from 21.7 to 80% at Wallaga University Referral Hospital (WURH) from January 1, 2023 to June 30, 2023.

**Methods:**

Hospital based interventional study was conducted at WURH. The Plan- Do-Study-Act (PDSA) cycle was used to test change ideas. A fishbone diagram and a driver diagram were used to identify root causes and address them. Major interventions included training of health professionals, initiation of pain as fifth vital sign, policy and protocol development, and conducting regular supportive supervision.

**Results:**

Upon completion of the project, overall pain-free hospital implementation increased from baseline 21.7–88.7%. Implementation of pain as 5th the vital sign was increased from 15.4 to 92.3%. Regular audits of pain assessment and management increased from 27.3 to 81.8%. Two standardized treatment protocols or chronic and acute pains were developed from baseline zero. A focal person for Pain-free hospital implementation was assigned. More than 85% of healthcare providers were trained in pain assessment and management.

**Conclusion:**

Compliance with pain-free hospital implementations was significantly improved in the study area. This was achieved through the application of multidimensional change ideas related to health professionals, standardized guidelines and protocols, supplies, and leadership. Therefore, we recommend providing regular technical updates & conducting a frequent clinical audit on pain management.

## Introduction

Pain is a distressing sensory and emotional sensation that is connected to, or similar to, existing or potential tissue injury. It can be classified based on its time course as either acute or chronic. Acute pain has an abrupt onset and may last up to 6 months if poorly managed [1–3].

Every person experiences pain differently, and biological, psychological, and social variables all have an impact. People come to understand the concept of pain as a result of their experiences in life. It is important to respect someone’s right to describe something as painful [1, 2, 4].

The American Pain Society has designated pain as the fifth vital sign due to its significant prevalence and suffering in an effort to enhance awareness of pain management among medical professionals, improve patient care, and increase the likelihood that patients will receive effective treatment [5, 6].

The majority of patients report pain, which is one of the most prevalent symptoms [1, 2]. Assessing the patients’ pain before and after an intervention is one of the pain management techniques [1, 6]. There are different factors that affect pain management [5, 7, 8]. Any healthcare system has three main obstacles that are related to patients, healthcare facilities, and healthcare staff [1, 8–10].

Developing countries tend to prioritize the eradication of poverty and hunger and the reduction of maternal and child mortality and pay little attention to pain management [5]. However, the Ethiopian Federal Ministry of Health (FMOH) has launched the Pain-Free Hospital Initiative (PFHI) in 2014 where pain management was integrated to other services. Still, pain management needs attention in different health facilities [7, 8]. Similarly, Wallaga University Referral Hospital has integrated pain management with other services. However, it was not effective due to factors related to training, patient education, leadership, and health professional concerns. This resulted in increased patient suffering, low patient satisfaction, and a negative hospital experience. Because of this, the Hospital Quality Improvement Team conducted a baseline survey on pain-free hospital implementations and identified low compliance.

### What is already known on this topic

Pain is one of the most common reasons people go to health facilities. However, in resource-limited settings, pain management is inadequate and not in line with international recommendations and standards.

#### What this study adds

After defining the root causes of low compliance with pain-free hospital implementation at Wallaga University Referral Hospital, an intervention plan was introduced involving a multidisciplinary team led by a quality expert and applying the Plan-Do-Study-Act cycle. The intervention succeeded in increasing compliance with pain-free hospital implementation. This leads to improved quality of care and a positive hospital experience.

#### How this study might affect research, practice or policy

This project highlights the positive impact of supportive supervision in implementing multidimensional interventions to improve the quality of health care.

## Methods and materials

### Study setting and period

This quality improvement project on pain-free hospital implementation is conducted at Wallaga University Referral Hospital from January 1, 2023, to June 30, 2023. Wallaga University Referral Hospital is the only teaching referral hospital in western Ethiopia. It is located in Nekemte town. The mission of the hospital is the provision of comprehensive medical care, health science training, and problem-solving research and interventions. It has a well-organized multidisciplinary Quality Support and Mentoring Team (QSMT) comprising physicians, nurses, pharmacists, laboratory technologists, anesthetists, and midwifery professionals.

This project was conducted by a multidisciplinary team (MDT) from the quality improvement unit, anesthesia, school of medicine, nursing, and pharmacy departments. The team consists of 2 senior physicians (1 anesthesiologist, 1 emergency critical care medicine specialist), 7 different professionals from the quality improvement unit (1 general practitioner, 1 pharmacist, 1 laboratory technologist, 1 midwifery professional, 4 nurse professionals), and 1 nurse. It was led by the clinical quality coordinator.

### Study design

A hospital-based multi-dimensional interventional study was conducted.

### Data collection and analysis

Based on a high patient load with moderate to severe pain, five departments (surgery and orthopedics, internal medicine, pediatrics, emergency and critical care, and oncology) were randomly selected. Data were collected by three trained data collectors using checklists adopted from reviewed literature [11–16]. Physical observations (like the presence of pain as the 5th vital sign, protocols, meeting agendas, and letter of focal person assignment) were carried out by the project members. In addition, supportive supervision was conducted by the quality team, project team, and department coordinators. The collected data were checked for completeness. It was then imported into an Excel database. The summary page received the computed and relevant outcome measures. The results were displayed on a run chart. We evaluate if an enhanced level of performance has been attained and is being maintained after each PDSA cycle.

## Baseline data

Before the implementation of the project, a baseline survey of five departments (surgery and orthopedics, internal medicine, pediatrics, emergency and critical care, and oncology) was conducted. From each department, 50 charts (10 from each department) were randomly selected. Additionally, 25 patients (5 patients from each department), and 25 health professionals (5 from each department) were randomly selected for interviews. Service sites were also observed for pain as the 5th the vital sign, presence of meeting agenda and treatment protocol, and the letter of the assigned focal person. Accordingly, the baseline data were.

- Compliance with pain-free hospital implementations was 21.7%.
- Compliance with pain as 5th the vital sign was 15.4%.
- Regular audits of pain assessment and management were 27.3%.
- Standardized pain treatment protocol was zero.
- No assigned focal person for pain assessment and management.

## Measurement

Different pain assessment and measurement tools were adopted from trusted sources. These are the WHO analgesic ladder, Wong-Baker Scale, numerical pain scale, FLACC scale, PAINAD scale, NIPS scale (neonatal infant pain scale), CRIES scale, behavioral pain scale, and critical care pain observation tool [11–16].

### Outcome measurement

- Proportion of pain-free hospital implementation at Wallaga University Referral Hospital.

### Process measures

- Proportion of health professionals who took training on pain assessment and its management.
- Proportion of proper implementation of pain as the 5th the vital sign.
- Proportion of patients assessed for pain.
- Proportion of available written protocols for acute and chronic pain management.
- Number of assigned focal person for pain-free hospital.
- Proportion of regular audits of pain assessment and management practices.

### Balancing measures

- Percentage of unnecessary pain medication given for patients.
- Frequent alerts, meetings and extra work to interns, residents, nurses, and physicians lead to increased work load.
- Number of works overloaded on staffs causing boring of staffs.
- Availing of different formats, posters, protocols, and management guidelines requires financial and facility cost.

## Strategy to impelement the project

**Fig. 1:**
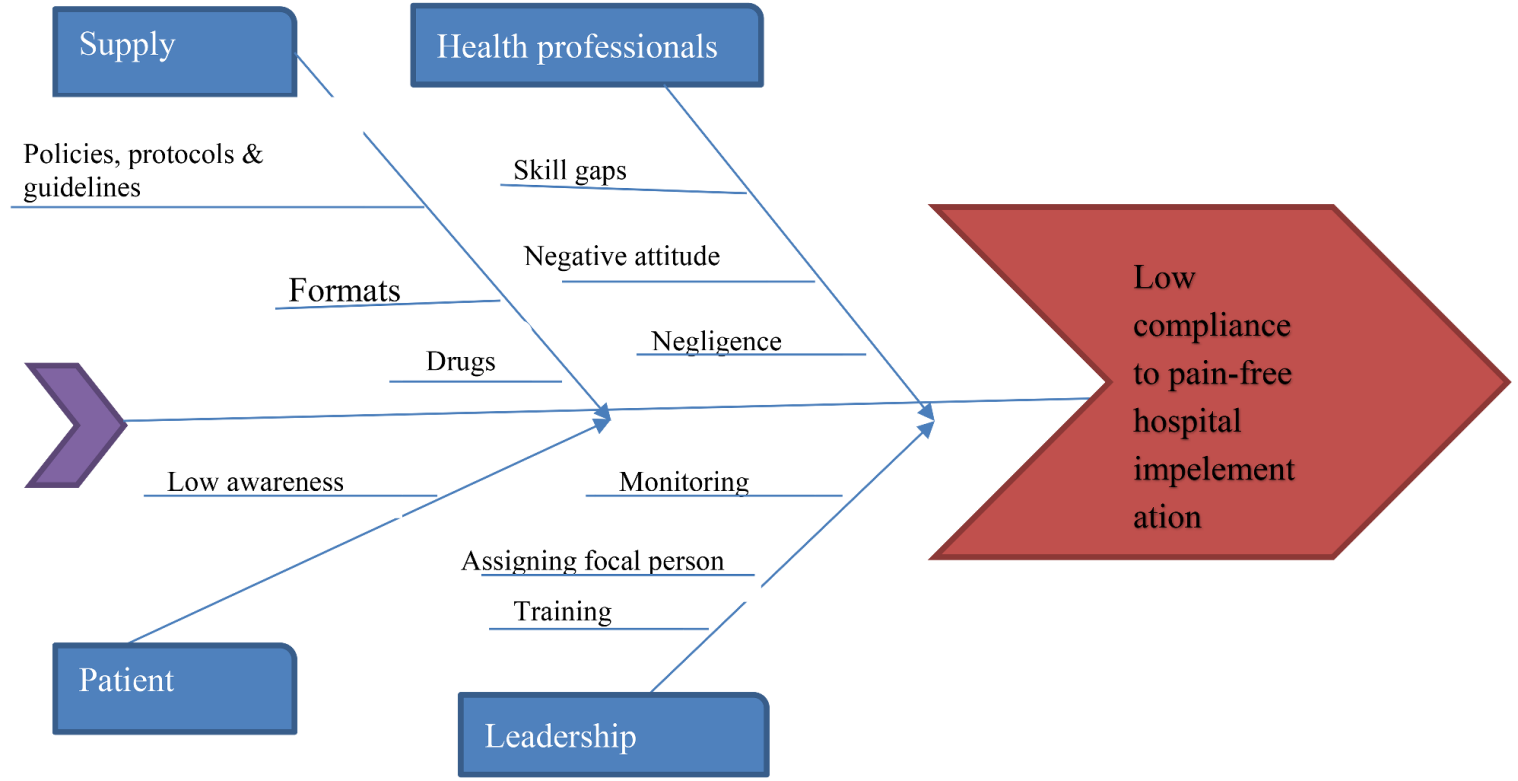
A fishbone diagram identifying and analyzing potential contributing root causes for low compliance to pain-free hospital implementation at wallaga university referral hospital, 2023

The MDT analyzed the root causes using a fishbone diagram (Fig. 1), plotted possible intervention packages, and designed an implementation plan. Six PDSA cycles were completed over 20 weeks. In each cycle, an intervention was implemented and studied for four weeks. Results were analyzed, and feedback was taken from multidisciplinary teams. Further interventions were explored in subsequent PDSA cycles, along with reinforcement of the previous one.

### Root cause analysis

In this study, using a fishbone diagram, the root causes of the problem were identified. The identified causes were inadequate training for health care professionals, a lack of written protocols and guidelines, the availability of medications at service points, no assigned hospital pain-free focal person or team, no regular audit on pain assessment and management, not recognizing pain as the 5th the vital sign, weak regular monitoring and evaluation from head nurses and department heads, and no patient education on pain and its management (Fig. 1).

### Interventions and change ideas

The following change ideas were targeted to increase the rate of pain management from baseline 21.7–80%. Proposed intervention and change ideas were.

- Onsite refreshment training for all healthcare professionals on pain assessment and its management to improve skill gaps and attitudes.
- Preparing standardized treatment policies, guidelines, and protocols for the management of acute and chronic pain.
- Implement pain as the fifth vital sign.
- Patient education on how to report pain and utilize pain medication.
- Assigning a focal person for pain management who closely works with the quality team.
- Availing medications for pain management.
- Regular coaching, mentoring, and supervision on pain assessment and management.

**Fig. 2:**
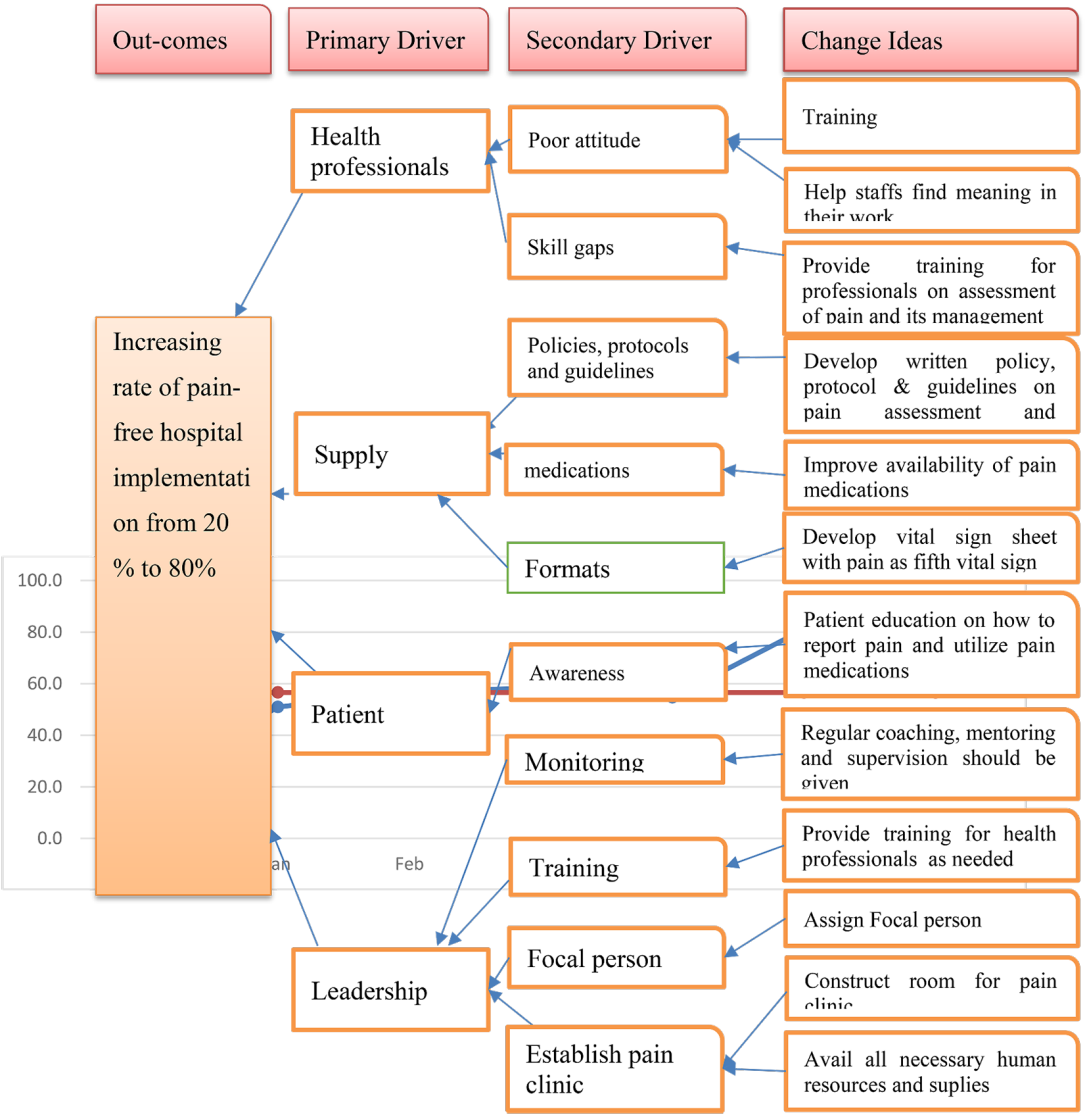
Driver diagram for increasing pain-free hospital Implementation rate at wallaga university referral hospital, 2023

Depending on the root causes identified (Fig. 1), four primary drivers, 10 secondary drivers, and 12 change or intervention ideas were schemed to achieve a pain-free hospital environment (Fig. 2).

## PDSA cycle of the project

### PDSA cycle 1

In the first PDSA cycle (sample size = 27), we wanted to identify major gaps in pain assessment and management at Wallaga University Referral Hospital using the World Health Organization (WHO) pain-free hospital implementation checklist. With our assessment, we identified major factors related to health professionals’ skills and attitudes, protocols and guidelines, patient awareness and drug utilization, and supportive supervision. We found that compliance with the hospital’s pain assessment and management was 50.9%.

With this finding, we also introduced the project to hospital management, different units, residents, and medical interns. Finally, we agreed on the low compliance and identified gaps and future plans to address the issue.

### PDSA cycle 2

In this cycle (sample = 30), we gave two days of training on the National Pain Management manual, the Pain Assessment Checklist, paint education on pain and its management, and the WHO Analgesic Ladder to 81% of health care professionals to address the skill gaps and negative attitude toward pain-free hospital implementation. This improved health professional awareness and attitude towards pain-free hospital implementation. We also highlighted that it is essential for all healthcare professionals to play their part to ensure pain-free hospital implementation.

Compliance with pain-free hospital implementation is 56.6%.

### PDSA cycle 3

In this cycle (sample size = 31), the MDT discussed with the hospital management the importance of assigning a focal person for pain-free hospital implementation who regularly supervises the progress of the project. Accordingly, the chief clinical director of the hospital has assigned a focal person for the successful implementation of this project. The focal person was given a clear orientation and job description, which were prepared based on the WHO checklist.

Compliance with pain-free hospital implementation is 58.6%.

### PDSA cycle 4

In this cycle (sample size = 29), we reinforced all previous interventions. The project team has developed essential documents, including pain follow-up formats (pain as the 5th the vital sign) and protocols. After we get approval of these documents from hospital management, staffs were oriented on how to use them.

Compliance with pain-free hospital implementation is 54.7%. It means there is slight decline from the previous cycle. This is because the project team has decreased the frequency of supportive supervision thinking that availability of formats and protocols alone might be enough.

### PDSA cycle 5

Given the previous PDSA cycle results (sample size = 37), the project team has provided the service units with essential pain medications. The health professionals were instructed on how and when to use the drugs. They were also advised of the importance of patient engagement in pain management.

Compliance with pain-free hospital implementation is 81.1%.

### PDSA cycle 6

In the last PDSA cycle (sample size = 37), we reinforced all previous interventions and conducted weekly supportive supervision on pain-free hospital implementations. During the follow-up, we identified some gaps among some health professionals and addressed them immediately.

In the 6th PDSA cycle, compliance with hospital pain free proper implementations is 88.7%.

## Results

**Fig. 3:**
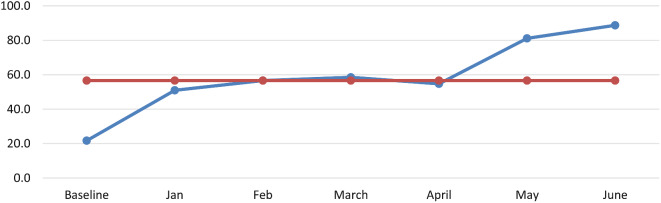
Run chart indicating pain-free hospital implementation rate at wallaga university referral hospital, 2023

Over all, the pain-free hospital implementation results during baseline and after project intervention were 21.7% and 88.7%, respectively, at Wallaga University referral hospital (Fig. 3). Implementation of pain as the fifth vital sign during baseline assessment was 15.4% and 92.3% after project implementation. Two standardized protocols on chronic and acute pain management for adults and children were developed. The focal person for pain-free hospital implementation was assigned by the CCD.

The regular audit of pain assessment and management practices was 27.3% during baseline assessment and has currently increased to 81.8%. 81% of health professionals trained in pain assessment and management from different departments.

## Discussions

This is a quality improvement project focused on improving compliance with pain-free hospital implementation by introducing change ideas. One of the interventions we made was related to health professionals. This is because different studies have clearly stated that health professionals’ knowledge, attitude, and practice towards pain assessment and management are crucial to implementing the pain-free hospital initiative [1, 5, 9]. Thus, in our project, we provided trainings on different aspects of pain for health professionals in the hospital.

Regular pain assessment and documentation are essential to achieving sufficient pain relief and thus improving the quality of care [1, 2, 5]. However, in this project, one of the root causes of low compliance was related to inappropriate pain assessment and documentation. This was because of a low level of awareness among health professionals and weak supportive supervision from the multidisciplinary team of the hospital.

Another important element for the implementation of a pain-free hospital is the availability of management protocols and drugs [6, 8, 11, 12]. Here, after we identified a lack of protocol and a limited variety and amount of pain medications, we worked with the hospital management and availed the drugs. We also demonstrated to the care providers how to use them and prevent the opioid epidemic, which is causing different crises globally [17]. Therefore, the hospital should stick to rational use of opioids based on the WHO analgesic ladder [16, 17].

## Limitations

In this project, the team focused on integrated pain-free hospital implementation with other services. We didn’t establish a separate pain clinic.

## Conclusions

The compliance with pain-free hospital implementations was significantly improved in the study area. This was achieved through the application of multidimensional change ideas related to health professionals, standardized guidelines & protocols, supplies, and leadership. Therefore, we recommend providing regular technical updates and conducting a frequent clinical audit on pain management.

## Data Availability

The data sets used and analyzed during the current study are available from the corresponding author on formal request.

## Abbreviations and acronyms

CCD: Chief Clinical Director
FMOH: Federal Ministry of Health
MDT: Multidisciplinary Team
PDSA: Plan- Do-Study-Act
PFHI: Pain-Free Hospital Initiative
QSMT: Quality Support and Mentoring Team
WHO: World Health Organization
